# Evaluating the effects of ivacaftor exposure on *Staphylococcus aureus* small colony variant development and antibiotic tolerance

**DOI:** 10.1093/jacamr/dlae185

**Published:** 2024-11-20

**Authors:** Gretchen E Bollar, Kendall M Shaffer, Johnathan D Keith, Ashley M Oden, Alexander E Dowell, Kevin J Ryan, Edward P Acosta, Jennifer S Guimbellot, Megan R Kiedrowski, Susan E Birket

**Affiliations:** Department of Medicine, Division of Pulmonary, Allergy, and Critical Care Medicine, University of Alabama at Birmingham, Birmingham, AL, USA; Gregory Fleming James Cystic Fibrosis Research Center, University of Alabama at Birmingham, Birmingham, AL, USA; Department of Medicine, Division of Pulmonary, Allergy, and Critical Care Medicine, University of Alabama at Birmingham, Birmingham, AL, USA; Gregory Fleming James Cystic Fibrosis Research Center, University of Alabama at Birmingham, Birmingham, AL, USA; Gregory Fleming James Cystic Fibrosis Research Center, University of Alabama at Birmingham, Birmingham, AL, USA; Department of Medicine, Division of Pulmonary, Allergy, and Critical Care Medicine, University of Alabama at Birmingham, Birmingham, AL, USA; Department of Pharmacology and Toxicology, Division of Clinical Pharmacology, University of Alabama at Birmingham, Birmingham, AL, USA; Department of Pharmacology and Toxicology, Division of Clinical Pharmacology, University of Alabama at Birmingham, Birmingham, AL, USA; Department of Pharmacology and Toxicology, Division of Clinical Pharmacology, University of Alabama at Birmingham, Birmingham, AL, USA; Gregory Fleming James Cystic Fibrosis Research Center, University of Alabama at Birmingham, Birmingham, AL, USA; Department of Pediatrics, Division of Pediatric Pulmonary and Sleep Medicine, University of Alabama at Birmingham, Birmingham, AL, USA; Department of Medicine, Division of Pulmonary, Allergy, and Critical Care Medicine, University of Alabama at Birmingham, Birmingham, AL, USA; Gregory Fleming James Cystic Fibrosis Research Center, University of Alabama at Birmingham, Birmingham, AL, USA; Department of Medicine, Division of Pulmonary, Allergy, and Critical Care Medicine, University of Alabama at Birmingham, Birmingham, AL, USA; Gregory Fleming James Cystic Fibrosis Research Center, University of Alabama at Birmingham, Birmingham, AL, USA

## Abstract

**Background:**

Ivacaftor exhibits anti-staphylococcal properties but does not clear *Staphylococcus aureus* from the lungs of people with cystic fibrosis (pwCF). We assessed whether exposure to therapeutic concentrations of ivacaftor could allow *S. aureus* to form small colony variants (SCVs), a phenotype commonly associated with bacterial persistence.

**Methods:**

Humanized G551D-CFTR (hG551D) rats were treated with ivacaftor for 7 days. Concentrations in the plasma, epithelial lining fluid and lung tissue lysate were measured using LC-MS/MS. Survival of *S. aureus* during ivacaftor treatment was assessed in an hG551D rat model of lung infection. *S. aureus* adaptation to therapeutic concentrations of ivacaftor was investigated *in vitro* by serial passage in the presence of 10 µM ivacaftor. Bacterial survival in the presence of antimicrobials was evaluated using growth curves and density assays.

**Results:**

Ivacaftor plasma concentrations of treated hG551D rats reached 3.488 ± 1.118 µM, with more variable concentrations in the epithelial lining fluid and lung tissue lysate. During *S. aureus* infection, ivacaftor-treated hG551D rats returned similar numbers of bacteria from the lung, compared with vehicle-treated controls. Exposure of *S. aureus* to ivacaftor *in vitro* led to the formation of ivacaftor-tolerant SCVs with an unstable phenotype and increased antibiotic tolerance.

**Conclusions:**

Treatment with ivacaftor did not alter *S. aureus* burden in the cystic fibrosis rat and led to the formation of tolerant SCVs *in vitro*, suggesting that development of an SCV phenotype may allow *S. aureus* to persist in the cystic fibrosis lung during ivacaftor therapy.

## Introduction

Cystic fibrosis (CF) is an autosomal recessive genetic disease caused by mutations in the gene encoding the CF conductance regulator (CFTR) anion channel protein.^1^ The absence of CFTR function in the airway leads to mucus stasis, inflammation and chronic infection, which often cause severe lung damage and eventual respiratory failure.^2–4^ The recent introduction of highly effective CFTR modulator therapy (HEMT) has significantly improved respiratory outcomes and increased life expectancy for many people with CF (pwCF).^5,6^ However, airway infection remains a major focus in CF treatment. *Staphylococcus aureus* is currently the most prevalent microorganism in the CF lung, and though the rate of colonization with some pathogens has declined with the use of HEMT, rates of *S. aureus* remain consistent in pwCF.^7–9^

With the approval the elexacaftor/tezacaftor/ivacaftor (ETI) triple modulator combination in 2019, HEMT options now cover approximately 90% of CFTR mutations.^7^ Notably, all HEMT combinations include ivacaftor, a potentiator of G551D-CFTR.^5,10–12^ Ivacaftor has been shown to reduce the lung burden of *Pseudomonas aeruginosa*, but does not affect the burden of MRSA or MSSA after 1 year of treatment.^8^ This is surprising, as ivacaftor has exhibited antistaphylococcal activity both *in vitro* and in murine models of other diseases.^13–16^

To investigate possible causes for *S. aureus* survival in pwCF, we modelled airway infection with *S. aureus* in the context of ivacaftor treatment using the hG551D-CFTR rat, which is responsive to ivacaftor.^17,18^ We also evaluated the response of *S. aureus* to ivacaftor exposure *in vitro*, with a focus on potential formation of small colony variants (SCVs). SCVs are slow-growing mutants of *S. aureus* that present with a colony size at least 10 times smaller than normal colony variants (NCVs), and are typically deficient in electron transport or DNA synthesis.^19,20^ These variants commonly exhibit increased antibiotic resistance, and have been associated with the use of antibiotics in the context of CF.^21–24^ Because exposure to multiple classes of antibiotics have led to the formation of resistant *S. aureus* SCVs *in vitro*,^25,26^ we hypothesized that exposure of *S. aureus* to ivacaftor may also lead to the generation of SCVs better suited to survive in the context of ivacaftor therapy.

## Materials and methods

### hG551D CF rat model

All animal experiments at the University of Alabama at Birmingham (UAB) were conducted in accordance with UAB Institutional Animal Care and Use Committee (IACUC)-approved protocols. Humanized-G551D-CFTR rats were used for all animal studies. Pups were generated by pairing heterozygote male and female rats and litters remained with lactating dams until weaning at 21 days of age. Homozygous G551D (termed hG551D) and WT littermates (termed WT) were used for experiments.^27^ WT and hG551D littermates were cohoused from weaning in standard cages kept at 21°C to 23°C with a 12 h light/dark cycle. Rats were given *ad libitum* access to a standard rodent diet and water supplemented with Pedialyte (Abbott, Abbott Park, IL, USA) reconstituted to manufacturer specifications, 12% polyethylene glycol and 0.1% sodium benzoate to reduce mortality from gastrointestinal obstruction.^28^ Animals were at least 6 months of age at study onset, with even numbers of male and female rats.

### Pharmacokinetic (PK) analysis of ivacaftor in the hG551D CF rat

*T*_max_ for ivacaftor in the plasma was predicted to be 1 h in rats via allometric scaling, as described by Caldwell *et al.* based on a *T*_max_ of 4 h and a terminal plasma half-life of 12 h in humans.^29,30^ hG551D rats were treated by oral gavage for with 40 mg/kg/day of ivacaftor (Selleck Chemicals, Houston, TX, USA) in 3% methylcellulose for 7 days and euthanized 1 h following the final dose by intraperitoneal injection of 500 µL of pentobarbital sodium (390 mg/mL). The thoracic cavity was opened and whole blood was collected from the vena cava and centrifuged at 4°C to separate plasma. Bronchoalveolar lavage fluid (BALF) was collected by tracheal cannulation and lavage with 5 mL of cold PBS (Thermo Fisher Scientific, Waltham, MA, USA). Blood was perfused from the tissue by injecting PBS into the heart until the lungs were cleared, and lungs were removed from the body. In preparation for ivacaftor concentration analysis, the right upper lobe was mechanically homogenized in Ham’s F-12 nutrient mix (Thermo Fisher Scientific). Cells were pelleted from lung homogenate via centrifugation and resuspended in 500 µL of NP-40 lysis buffer (Thermo Fisher Scientific). Ivacaftor concentration in the plasma, BALF and lung cell lysate were determined using LC with tandem MS (LC-MS/MS), validated using ivacaftor reference standards as recommended by the FDA for PK studies.^31,32^ Concentrations of urea in the plasma and BALF were determined via colorimetric assay (Urea Colorimetric Assay Kits II and III, Millipore Sigma, St. Louis, MO, USA). Concentration of ivacaftor in epithelial lining fluid (ELF) was corrected by comparing the ratio of urea in the BALF to that in plasma, as described by Rennard *et al*.^33^

### Bacterial strains used

The MRSA strain SA0831 was cultured at UAB from an adult inpatient with CF, chronically infected with *S. aureus* and *P. aeruginosa* and tested for antibiotic tolerance, as previously described.^34^ SA0831^iva^ is derived from SA0831 as detailed below.

### Bacterial growth and maintenance

Prior to use in all assays, SA0831 was inoculated onto mannitol salt agar (MSA) and incubated overnight at 37°C. SA0831^iva^ was inoculated onto MSA supplemented with 10 µM ivacaftor and incubated for 72 h at 37°C. For *in vitro* assays, liquid cultures of SA031 and SA0831^iva^ were grown overnight at 37°C with shaking in double-concentrated brain heart infusion broth (2× BHI) or 2× BHI supplemented with 10 µM ivacaftor, respectively. Liquid cultures were washed with PBS and resuspended in the appropriate media as indicated for each assay.

### *S. aureus* infection and ivacaftor treatment

SA0831 was grown overnight at 37°C with shaking in 1× BHI, washed with PBS, and resuspended to a 10^9^ cfu/300 µL dose in PBS. On study Day 0, WT and hG551D rats were anaesthetized by isoflurane and *S. aureus* was instilled intratracheally. Cfu in inoculum were confirmed by plating serial dilutions onto MSA and manually counting colonies after incubation for ≥24 h at 37°C. Seventy-two hours after infection, rats were administered 3% methylcellulose vehicle or 40 mg/kg ivacaftor in 3% methylcellulose by oral gavage.^27^ Rats were subsequently treated with ivacaftor or vehicle on Days 4, 5 and 6, with 24 h between doses. Weight was monitored daily. Animals were euthanized by intraperitoneal injection of 500 µL of pentobarbital sodium (390 mg/mL) and exsanguination 24 h after the final dose. BALF was collected by tracheal cannulation and lavage with 5.0 mL of cold PBS and the thoracic cavity was opened for tissue collection. Lungs were removed and each lobe was homogenized separately in Ham’s F-12 nutrient mix. Serial dilutions of homogenate for each lobe were plated on MSA and incubated for up to 3 days at 37°C. Resulting colonies were counted and cfu/lobe were combined to yield total cfu/lung. Total numbers of rats for this experiment are as follows: WT + *S. aureus* + vehicle = 3 rats; hG551D + *S. aureus* + vehicle = 4 rats; and hG551D + *S. aureus* + ivacaftor = 4 rats.

### Genetic sequencing

Colonies from the lungs of the rats treated with ivacaftor were grown as described above and treated with enzymatic lysis buffer containing recombinant lysostaphin (Ambicin L, Ambi Products LLC, Lawrence, NY, USA) prior to genomic DNA extraction using the DNeasy blood and tissue kit spin-column protocol (QIAGEN, Hilden, Germany). Gene sequencing was performed by the microbial genome sequencing centre (SeqCenter; Pittsburgh, PA, USA) using a combination of long and short reads. Short-read sequencing was performed with an Illumina NextSeq2000 to generate 151 bp paired-end reads with 4 000 000 reads per sample. Long-read sequencing was conducted using Oxford Nanopore Technologies (ONT) sequencing, with 300 000 long reads per sample, having an average length of 5 kb each. Quality control and adapter trimming was performed by MiGS using bcl2fastq (v.2.20.0.445) for Illumina sequencing and Porechop (v. 0.2.3_seqan2.1.1) for ONT sequencing prior to genome assembly and annotation. Hybrid assembly of Illumina and ONT reads was performed by MiGS using the Unicycler package (v. 0.4.8), with statistics recorded with QUAST. Assembly annotation was performed by SeqCenter using Prokka. Variant calling between genomes was performed by MiGS using breseq (v. 0.35.4) to align and compare sequencing data. Further sequence analysis was performed using the PATRIC platform, and multilocus sequence type was determined using the MLST app in OmicsBox (version 2.1.14), as conducted previously.^34^

### Cytokine and mucin analysis

A bicinchoninic acid (BCA) protein assay kit (Millipore Sigma, St. Louis, MO, USA) was used to measure total protein content in the BALF. Levels of Muc5b and Muc5ac were detected using a modified Western blot technique for large-molecular-weight proteins on nitrocellulose membranes, with antibodies selective for each mucin as previously described.^18,35–37^ Horseradish peroxidase secondary antibodies were used with SuperSignal West Femto Maximum Sensitivity Substrate (Thermo Fisher Scientific) to detect protein signal. Blots were detected for chemiluminescence and analysed for densitometry using Fiji ImageJ software.^38^ Concentrations of IL-1β in the BALF were determined using a precoated sandwich ELISA kit per the manufacturer’s protocol (ab255730 Rat IL-1 beta ELISA Kit).

### Generation of an SCV *S. aureus* strain from ivacaftor exposure

SA0831 was grown from frozen stock for 24 h on MSA at 37°C, and a single colony was streaked over the surface of MSA supplemented with 10 µM ivacaftor. After 48 h incubation at 37°C, a single small colony was chosen and transferred to a new MSA plate with 10 µM ivacaftor. This process was repeated with subsequent colonies passaged on MSA with ivacaftor every 48–72 h, for a total of 10 passages, previously shown to reliably yield SCV phenotypes for other antibiotic passage schemes.^34,39^ Following the final passage, a single colony was chosen and transferred to liquid 1× BHI containing 10 µM ivacaftor and grown for 48 h with shaking at 37°C, washed with PBS, and used to create a frozen glycerol stock of SA0831^iva^ to be used in further assays. This scheme was also attempted with plates containing 100 µM ivacaftor, but did not result in viable bacterial colonies for passage.

### Bacterial growth curves and response to antibiotic exposure

Overnight liquid cultures of SA0831 and SA0831^iva^ were washed with PBS, resuspended in 1× BHI supplemented with ivacaftor and/or antibiotics, and seeded in 200 µL aliquots in 96-well plates at approximately 10^6^ cfu of bacteria per well. Inoculating cfu were confirmed by plating serial dilutions of inoculum on MSA and manually counting resulting colonies after incubation at 37°C for up to 3 days. The 96-well plates were incubated at 37°C for 24 h with continuous shaking and absorbance readings at 600 nm every 20 min. Conditions included no drug control, 10, 50 and 100 µM ivacaftor, 50 µg/mL tobramycin, 240 µg/mL trimethoprim/ sulfamethoxazole, 1 µg/mL linezolid, 10 µg/mL azithromycin or 10/1.25 µg/mL piperacillin/tazobactam. Antibiotics were tested alone or in combination with 10 µM ivacaftor. Compounds insoluble in water were reconstituted in DMSO (Sigma–Aldrich, St. Louis, MO, USA) prior to addition to BHI, with final DMSO concentrations in media at ≤1%, previously shown not to affect the growth of *S. aureus*.^34^ Growth curves are composites of three biological replicates with five technical replicates each. Cfu remaining after treatment were assessed by plating serial dilutions of culture from individual wells on MSA and manually counting colonies after incubation at 37°C for up to 3 days. Cfu analyses of bacterial survival were performed as three biological replicates with two technical replicates.

### Statistics

Statistical analysis was performed with GraphPad Prism version 9.5.1 (GraphPad Software, San Diego, CA, USA). For weight analysis, significance between groups was determined by two-way ANOVA, considering the overall effect of group when comparing each individual group to a single other group. For all other comparisons, significance between groups was computed via one-way ANOVA for normal data and the Kruskal–Wallis test for non-normal data, as determined by the Shapiro–Wilk test, with all groups included in analysis. For *in vitro* cfu fold-change analyses, *P* values are reported only for between-strain comparisons for each drug combination tested. Results of cfu analyses were cleaned for outlier wells using the ROUT method (Q = 1%). *P* < 0.05 was considered significant. Statistics are presented as mean ± SEM.

### Study approval

Collection of bacteria from patient sputum samples was approved by the UAB Institutional Review Board (IRB-160720008). Animal experiments were approved by the UAB IACUC (IACUC 22206).

## Results

### Evaluation of ivacaftor concentrations in the CF rat lung

**Figure 1.**
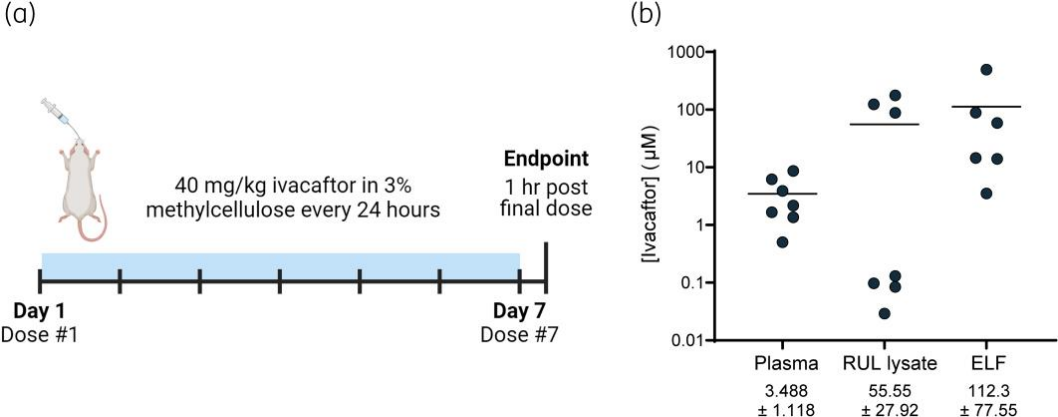
Ivacaftor concentrations in CF rat tissue. Uninfected hG551D rats >6 months of age were orally dosed with ivacaftor daily for 7 days, and were sacrificed 1 h following the final dose. (a) Graphical representation of timeline. Figure generated in Biorender. (b) Ivacaftor concentrations in lung homogenate from lung right upper lobe (RUL) lysate, plasma and ELF were determined by LC-MS/MS analysis.

To determine the amount of ivacaftor that bacteria may be exposed to in the lung, we conducted PK analysis of ivacaftor concentration in the airway cells, ELF and plasma. We treated hG551D rats daily with ivacaftor by oral gavage for 7 days, sacrificing 1 h following the final dose at projected plasma *T*_max_ (Figure 1a). Using an LC-MS/MS assay for detection of ivacaftor, we found the average concentration in the plasma to be 3.488 ± 1.118 µM (Figure 1b). Ivacaftor concentrations in lung tissue lysate and ELF were more variable, with an average of 55.55 ± 27.92 and 112.3 ± 77.55 µM, respectively. These values match what has been found in human studies of ivacaftor PK in the airway cells.^31,32^

### Effect of ivacaftor treatment on acute *S. aureus* lung infection in the hG551D rat

**Figure 2.**
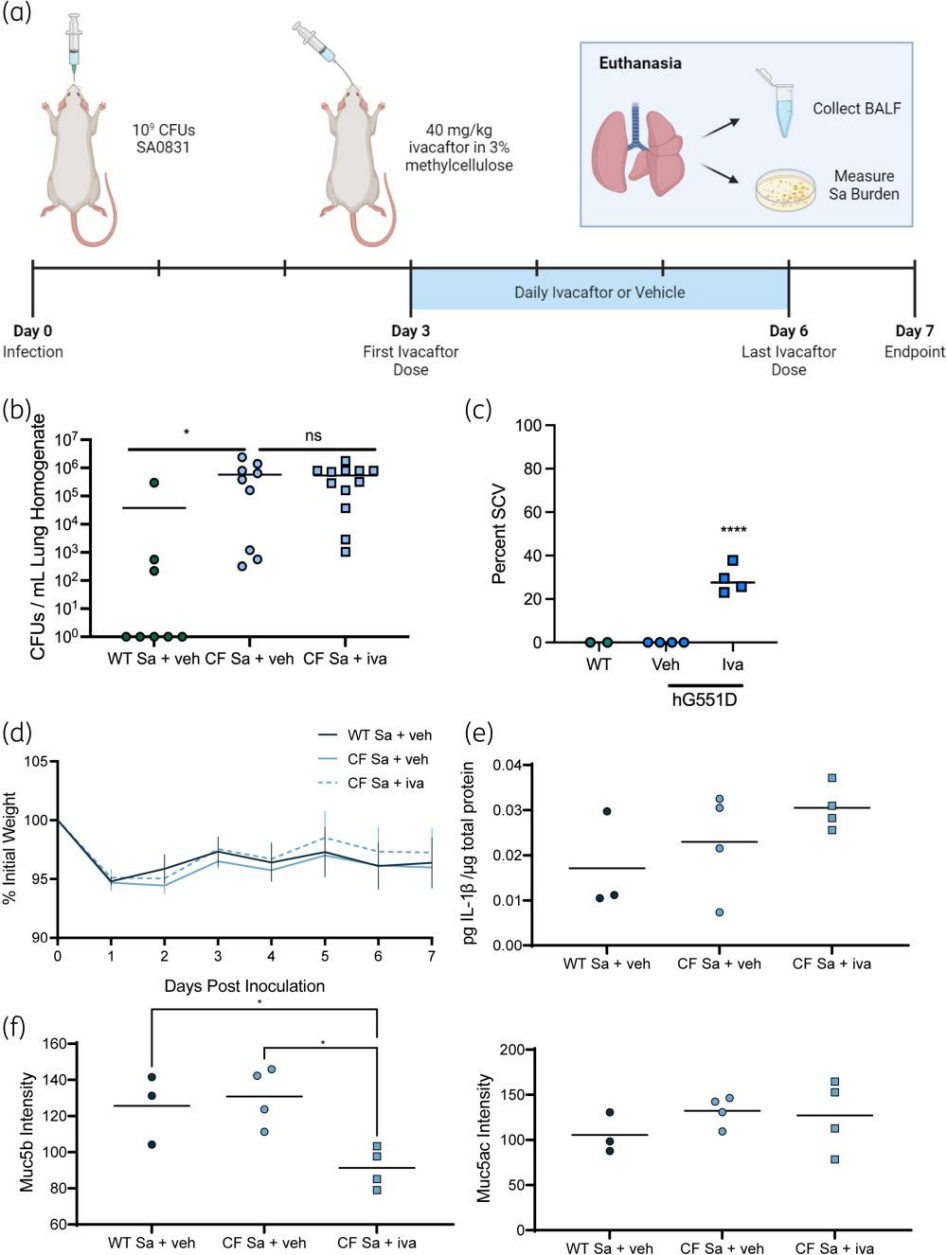
Ivacaftor treatment post-infection does not clear *S. aureus* SA0831 from the lungs of CF rats. WT and hG551D (CF) rats >6 months of age were infected intratracheally with 10^9^ cfu of *S. aureus* (Sa) strain SA0831, dosed daily with ivacaftor (iva) or vehicle (veh) on Days 3 to 6 post-infection, and were sacrificed on Day 7. (a) Graphical representation of timeline and endpoints. (b) Cfu of *S. aureus* in the lung were determined by plating lung homogenate on MSA. (c) Colonies were assessed by plating for NCV or SCV phenotypes, with percentage of colonies with the SCV phenotype reported. (d) Weight change was monitored over time relative to initial weight. (e) Amount of IL-1β in the BALF was measured via ELISA. Mucin content of (f) Muc5b and (g) Muc5ac in the BALF was determined by modified Western blot for large-molecular-weight proteins. *P ≤ 0.05, ****P < 0.0001.

We have previously demonstrated that *S. aureus* strain SA0831 is susceptible to growth inhibition by ivacaftor *in vitro*.^34^ To investigate whether ivacaftor mitigates *S. aureus* burden and pathology *in vivo*, we intratracheally infected WT or hG551D rats with 10^9^ cfu of SA0831. hG551D rats were treated with ivacaftor or methylcellulose vehicle, beginning on Day 3 post-infection (Figure 2a). We evaluated bacterial burden in the lung on Day 7 post-infection, 24 h following the final dose of ivacaftor. The burden of *S. aureus* was similar in the lung homogenates of both hG551D treatment groups (Figure 2b), indicating no significant effect of post-infection ivacaftor treatment on *S. aureus* survival. Lung homogenates were also grown in the presence of ivacaftor, which identified that approximately 25% of bacteria recovered from the lungs of ivacaftor-treated hG551D rats had the SCV phenotype (Figure 2c). Weight was monitored as a marker of disease severity, and though all groups dropped from their initial weight by Day 1, there was no significant difference between rats that received ivacaftor or vehicle (Figure 2d). No difference was observed in levels of the proinflammatory, promucin secretory cytokine IL-β (Figure 2e) in the BALF. Ivacaftor treatment did improve mucus burden in the lung, with lower Muc5b mucin levels in the BALF from the ivacaftor-treated hG551D rats compared with their vehicle-treated counterparts (Figure 2f), although there was no difference in levels of Muc5ac (Figure 2f). These data indicate that treatment with ivacaftor does not significantly reduce the host response to infection.

Colonies isolated from the lungs of hG551D rats treated with ivacaftor were assessed by WGS to determine if there were any genetic changes to the bacteria. Analysis of the SA0831 parent strain was conducted previously.^34^ Here we conducted variant calling of the bacteria collected from the ivacaftor-treated hG551D rat lungs using breseq. We found numerous SNPs associated with genes that have previously been associated with the development of the SCV phenotype (Table 1), with antibiotic tolerance (Table 2) and with both (Table 3) identified via the PATRIC platform. Interestingly, we found that both the menadione and the haemin pathways were affected, with mutations to several genes in each pathway.

**Table 1.** SCV-associated genes

Gene	Description
*accB_1*→	Biotin carboxyl carrier protein of acetyl-CoA carboxylase
*accD*→	Acetyl-CoA carboxylase carboxyl transferase subunit β
*aroA_1*→	Protein AroA(G)
*aroA_2*→	3-Phosphoshikimate 1-carboxyvinyltransferase
*aroB*→	3-Dehydroquinate synthase
*aroD*←	3-Dehydroquinate dehydratase
*aroE*→	Shikimate dehydrogenase [NADP(+)]
*aroK*→	Shikimate kinase
*ccpA*→	Catabolite control protein A
*citZ*→	Citrate synthase 2
*ctaA*→	Haem A synthase
*fabF*←	3-Oxoacyl-[acyl-carrier-protein] synthase 2
*fabH*←	3-Oxoacyl-[acyl-carrier-protein] synthase 3
*fumC*→	Fumarate hydratase class II
*hemC*→	Porphobilinogen deaminase
*menC*→	*o*-Succinylbenzoate synthase
*menD*←	2-Succinyl-5-enolpyruvyl-6-hydroxy-3-cyclohexene-1-carboxylate synthase
*menE_1*→	2-Succinylbenzoate–CoA ligase
*menE_2*←	2-Succinylbenzoate–CoA ligase
*menF*←	Isochorismate synthase MenF
*menH*←	2-Succinyl-6-hydroxy-2,4-cyclohexadiene-1-carboxylate synthase
*pyrD*←	Dihydroorotate dehydrogenase (quinone)
*qorB*→	Quinone oxidoreductase 2
*saeS*→	Histidine protein kinase SaeS
*scn_1*→	Staphylococcal complement inhibitor
*scn_3*→	Staphylococcal complement inhibitor
*sigB*→	RNA polymerase sigma factor B
*glpD*←	Aerobic glycerol-3-phosphate dehydrogenase
*vraR*→	Response regulator protein VraR
*vraS*→	Sensor protein VraS
*rpoC*←	DNA-directed RNA polymerase subunit β
*hrtA_1*←	Putative haemin import ATP-binding protein HrtA
*pdhC_1*→	Dihydrolipoyllysine-residue acetyltransferase component of pyruvate dehydrogenase complex
*rsbU*→	Phosphoserine phosphatase RsbU
*icd*→	Isocitrate dehydrogenase [NADP]
*gltX*←	Glutamate–tRNA ligase
*nupC_2*←	Nucleoside permease NupC
*hemL1*→	Glutamate-1-semialdehyde 2,1-aminomutase 1
*hemL2*←	Glutamate-1-semialdehyde 2,1-aminomutase 2
*hemW*→	Haem chaperone HemW
*hemY*→	Protoporphyrinogen oxidase

Summary of genetic changes associated with the SCV phenotype in SA0831^iva^ compared with the parent SA0831, as determined by breseq. → indicates a downstream frameshift; ← indicates an upstream frameshift.

**Table 2.** Antibiotic tolerance-associated genes

Gene	Description
*bcr_2*→	Bicyclomycin resistance protein
*gyrA*←	DNA gyrase subunit A
*gyrB*←	DNA gyrase subunit B
*lnrL_2*→	Linearmycin resistance ATP-binding protein LnrL
*mepA*←	Multidrug export protein MepA
*pbpH*→	PBP H
*ponA*←	PBP 1A/1B
*tet*(38)←	Tetracycline efflux MFS transporter Tet(38)
*tet*(A)←	Tetracycline resistance protein, class B
*emrY*→	Putative multidrug resistance protein EmrY
*mdtD*→	Putative multidrug resistance protein MdtD
*yhfP*←	Putative quinone oxidoreductase YhfP
*qoxB*→	Putative quinol oxidase subunit 1

Summary of genetic changes associated with antibiotic tolerance in SA0831^iva^ compared with the parent SA0831, as determined by breseq. → indicates a downstream frameshift; ← indicates an upstream frameshift.

**Table 3.** SCV and antibiotic-tolerance genes

Gene	Description
*agrA*←	Accessory gene regulator protein A
*agrB*←	Accessory gene regulator protein B
*ndhB*←	NAD(P)H-quinone oxidoreductase subunit 2, chloroplastic
*norB_1*→	Quinolone resistance protein NorB
*norB_2*→	Quinolone resistance protein NorB
*norB_3*←	Quinolone resistance protein NorB
*norB_4*→	Quinolone resistance protein NorB
*fabF*←	3-Oxoacyl-[acyl-carrier-protein] synthase 2
*mqo1*→	Putative malate:quinone oxidoreductase 1

Summary of genetic changes associated with both the SCV phenotype and antibiotic tolerance in SA0831^iva^ compared with the parent SA0831, as determined by breseq. → indicates a downstream frameshift; ← indicates an upstream frameshift.

### Formation of an unstable ivacaftor-driven SCV phenotype

**Figure 3.**
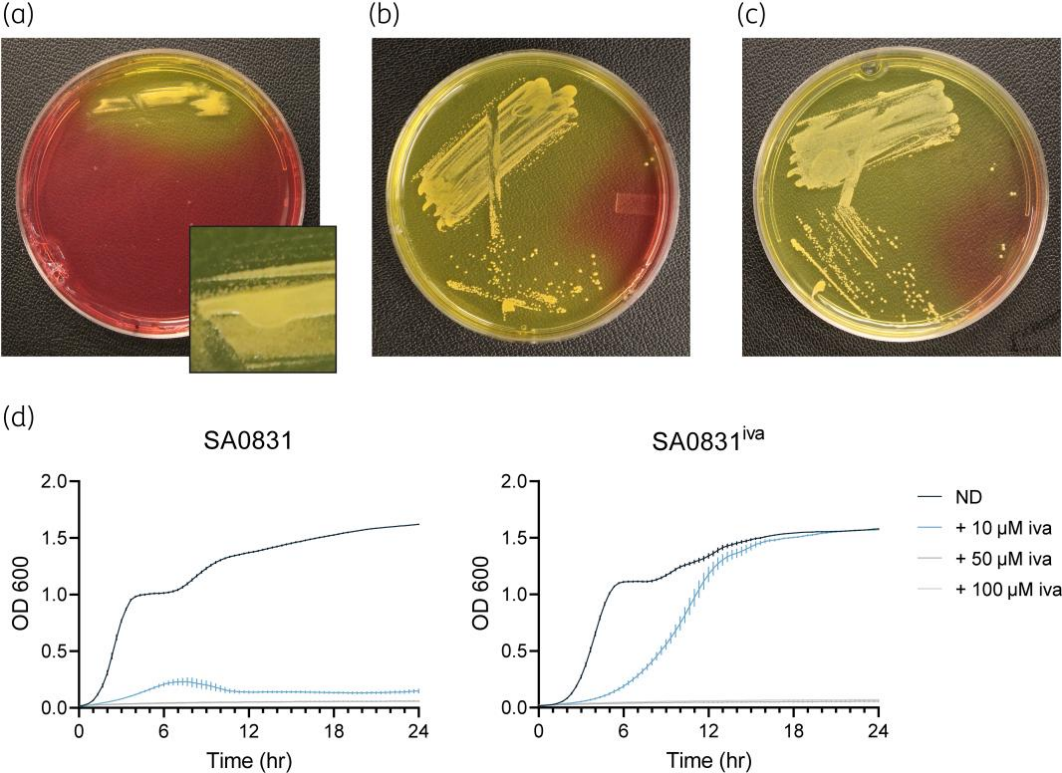
Exposure of *S. aureus* to ivacaftor *in vitro* led to the formation of an unstable SCV phenotype. (a) Representative images of the SA0831^iva^ mutant at 72 h growth on MSA supplemented with ivacaftor (iva) with inlay showing individual colonies; (b) parent strain SA0831 at 48 h growth on MSA; and (c) SA0831^iva^ at 48 h growth on MSA with a parental phenotype indicating reversion. (d) Growth was also evaluated for liquid cultures of SA0831 and SA0831^iva^ in BHI with no drug (ND) or supplemented with varying concentrations of ivacaftor.

SCVs of *S. aureus* are frequently associated with increased antibiotic resistance, generating the hypothesis that the development of this phenotype presents an advantage to bacterial survival during antibiotic treatment.^19^ To determine whether exposure to ivacaftor could alter the phenotype of *S. aureus* during the course of infection, we serially passaged SA0831 with 10 µM ivacaftor *in vitro*, and found that the presence of ivacaftor over a prolonged period of time resulted in the SCV phenotype. We selected a single colony from the final passage to use in further assays, hereafter referred to as SA0831^iva^. On MSA supplemented with ivacaftor, SA0831^iva^ was only visible after 3 days of growth with pinpoint colonies (Figure 3a), while the SA0831 parent strain was fully grown on MSA after 24 h of growth, with a larger colony size that was well-defined at 48 h (Figure 3b). However, when SA0831^iva^ was grown on MSA without ivacaftor, the growth phenotype matched that of the parent (Figure 3c). This indicates that *S. aureus* may shift to an SCV phenotype during ivacaftor exposure and is capable of rapid reversion to NCV when ivacaftor is not present. In liquid culture, SA0831^iva^ was also tolerant of exposure to 10 µM ivacaftor, whereas growth of SA0831 was inhibited at this concentration (Figure 3d). At concentrations above 10 µM, growth of both strains was inhibited.

### Prior exposure to ivacaftor increases antibiotic tolerance of SA0831

**Figure 4.**
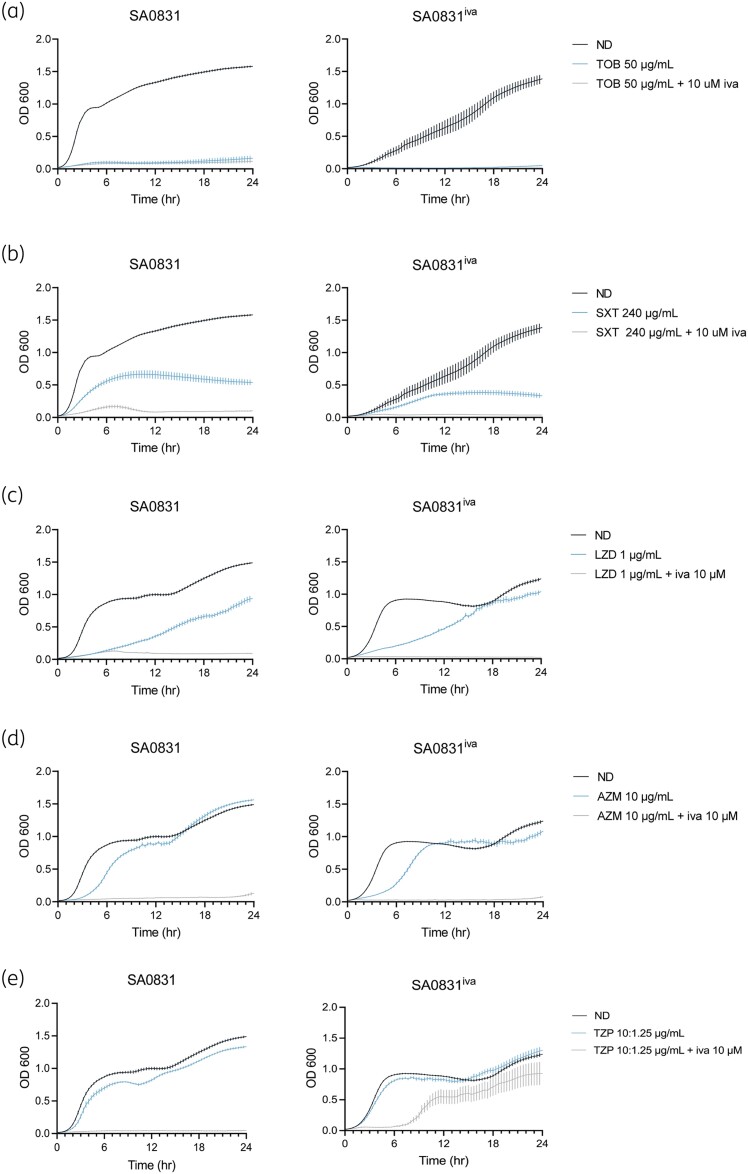
SA0831^iva^ exhibits a different antibiotic tolerance profile from its parent strain. Growth of SA0831 and SA0831^iva^ was measured over 24 h shaking in BHI supplemented with no drug (ND) or with (a) 50 μg/mL tobramycin (TOB) alone or in combination with 10 µM ivacaftor (iva), (b) 240 μg/mL trimethoprim/sulfamethoxazole (SXT) alone or in combination with 10 µM iva, (c) 1 μg/mL linezolid (LZD) alone or in combination with 10 µM iva, (d) 10 μg/mL azithromycin (AZM) alone or in combination with 10 µM iva and (e) 10/1.25 μg/mL piperacillin/tazobactam (TZP) alone or in combination with 10 µM iva.

Because SCVs have been shown to display increased resistance to multiple classes of antibiotics, we also evaluated growth of SA0831^iva^ during exposure to antibiotics commonly prescribed to pwCF in combination with ivacaftor. In liquid culture, no clear growth advantage of the SCV phenotype was seen for tobramycin (Figure 4a), trimethoprim/sulfamethoxazole (Figure 4b), linezolid (Figure 4c) or azithromycin (Figure 4d). However, while growth of SA0831 was inhibited by exposure to piperacillin/tazobactam in combination with ivacaftor, SA0831^iva^ reached a similar final OD as its untreated control (Figure 4e).

**Figure 5.**
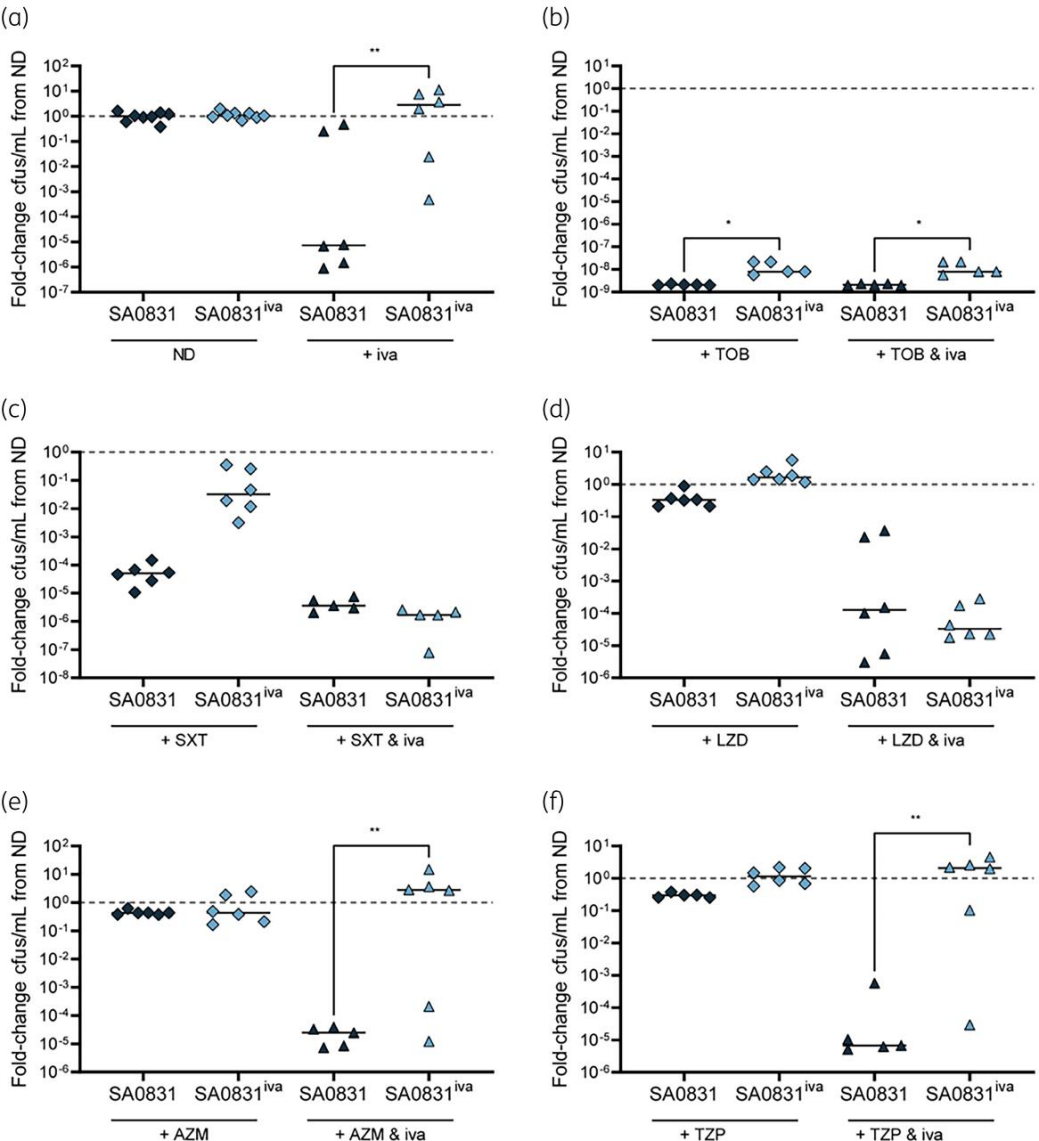
SA0831^iva^ exhibits increased persistence during antibiotic exposure compared with its parent strain. Growth of SA0831 and SA0831^iva^ was measured after 24 h shaking in BHI supplemented with (a) no drug (ND) or with 10 µM ivacaftor (iva), (b) 50 μg/mL tobramycin (TOB) alone or in combination with 10 µM iva, (c) 240 μg/mL trimethoprim/sulfamethoxazole (SXT) alone or in combination with 10 µM iva, (d) 1 μg/mL linezolid (LZD) alone or in combination with 10 µM iva, (e) 10 μg/mL azithromycin (AZM) alone or in combination with 10 µM iva and (f) 10/1.25 μg/mL piperacillin/tazobactam (TZP) alone or in combination with 10 µM iva. Fold-change is calculated respective to growth under the ND condition. *P* represents between-strain comparisons of fold-change under each condition. *P ≤ 0.05, **P < 0.01, ***P < 0.001, ****P < 0.001.

For these assays, we also investigated the amount of live bacteria present in these cultures after 24 h growth. Cfu of each strain, relative to untreated, were compared for each drug combination. We found that after exposure to ivacaftor alone, relative cfu of SA0831^iva^ were increased compared with those of SA0831 (Figure 5a). After tobramycin exposure, relative cfu of SA0831^iva^ were significantly higher than those of SA0831, with or without ivacaftor (Figure 5b). Conversely, relative cfu of SA0831 and SA0831^iva^ were similar after exposure to trimethoprim/sulfamethoxazole (Figure 5c) or linezolid (Figure 5d), with or without ivacaftor. Similar relative cfu were also recovered for SA0831 and SA0831^iva^ after exposure to azithromycin (Figure 5e) or piperacillin/tazobactam (Figure 5f), but the addition of ivacaftor in combination with either drug led to significantly increased relative cfu of SA0831^iva^ compared with SA0831. Taken together, these results indicate that the SA0831^iva^ mutant exhibits increased antibiotic tolerance compared with the NCV parent strain, especially in the context of ivacaftor exposure.

## Discussion

The presence of a quinoline ring in the chemical structure of ivacaftor has previously led to speculation regarding its potential as an antibacterial agent.^13^ Many quinoline derivatives have been shown to exhibit antimicrobial properties, with some being adapted for medical use as antibiotics such as ciprofloxacin.^40^ Studies investigating the antibacterial effects of ivacaftor have shown that ivacaftor exhibits antistaphylococcal activity *in vitro*^13–15^ and in a neutropenic thigh infection model *in vivo*.^16^ Despite this, *S. aureus* persists during ivacaftor treatment in CF, as pwCF treated with ivacaftor alone or in combination with elexacaftor and tezacaftor experience similar rates of colonization with *S. aureus* as before starting treatment.^8,9^ To better understand these differences in outcome, we assessed both the *in vivo* effects of ivacaftor on *S. aureus* survival in the hG551D rat model and evaluated phenotypic changes to *S. aureus* following ivacaftor exposure *in vitro*.

After 1 week of treatment with ivacaftor, levels in the plasma of uninfected hG551D rats were similar to data from human studies that report peak ivacaftor levels in the plasma of pwCF at 1390 ng/mL (3.541 µM) and 2385 ng/mL (6.077 µM).^32,41^ We have previously shown that treatment with ivacaftor alleviates mucus abnormalities in the uninfected hG551D rat by improving mucus transport and reducing the level of mucin proteins in the BALF.^18^ In this study, we found that ivacaftor administration to hG551D rats infected with *S. aureus* also led to a reduction in mucus protein levels in the BALF, despite similar promucin signalling via IL-1β.^42^ However, ivacaftor treatment did not lead to a reduction in bacterial burden in the lung. These results align with data from pwCF that indicate *S. aureus* persists in the CF lung during ivacaftor treatment, and highlight a more direct link between ivacaftor use and bacterial persistence by removing confounding clinical factors such as colonization with other pathogens and prior medical history.^8,9^

Phenotypic changes to *S. aureus* during ivacaftor exposure may contribute to its persistence in the CF lung. In the presence of environmental stressors such as antibiotics, *S. aureus* has been shown to form SCVs that exhibit enhanced survival capabilities at the expense of reduced growth rates and slower metabolism in comparison with NCVs.^19,26,43^ In the absence of selective pressure, these SCVs are frequently capable of switching back to an NCV phenotype, making them difficult to detect in culture.^44,45^ Studies show that *in vitro* exposure to quinolone antibiotics can lead to the formation of *S. aureus* SCVs, with some rapidly transitioning to NCVs upon subculture.^46–48^ Additionally, exposure to the quinolone *P. aeruginosa* exoproduct 2-heptyl-4-hydroxyquinoline-*N*-oxide (HQNO), has been shown to induce formation of reversion-capable *S. aureus* SCVs with increased antibacterial resistance.^49,50^ In this study, we showed that *in vitro* passage of *S. aureus* CF clinical isolate SA0831 with 10 µM ivacaftor led to the formation of the phenotypically unstable SCV SA0831^iva^, which reverted to NCVs phenotypically matching the parent when cultured on media without ivacaftor. Unlike its NCV parent, SA0831^iva^ is tolerant of ivacaftor *in vitro*. Because the SCV phenotype of SA0831^iva^ is only exhibited in the presence of ivacaftor, it is possible that similar mutations may occur in the CF lung during ivacaftor treatment, reverting to NCVs during laboratory culture and missing clinical detection as a result.

The ability of SCVs to transition between phenotypes may be beneficial to *S. aureus* as it colonizes the CF lung environment. In uninfected hG551D rats treated with ivacaftor for 1 week, we found that concentrations in the lung tissue lysate and ELF were variable. Similarly, in small cohort studies of pwCF, ivacaftor concentrations in volume-normalized nasal epithelial cell lysates ranged from 0 to 9.88 × 10^4^ ng/mL (252 µM).^31,32^ This suggests that during ivacaftor treatment, *S. aureus* may encounter differing amounts of ivacaftor in the lung and potentially shift to tolerant SCVs with reduced growth in areas of high exposure, and reverting to NCVs in areas of low exposure to restore normal growth. Previous studies have also reported additive or synergistic effects between ivacaftor and antibiotics against *S. aureus,* indicating that the addition of ivacaftor may be beneficial for killing *S. aureus* in the lung during antibacterial treatment.^13–15^ Here we found that the SA0831^iva^ mutant displayed increased persistence compared with its NCV parent during exposure to combinations of antibiotics and ivacaftor, although ivacaftor may be acting synergistically with some of the antibiotics tested. Accordingly, increased antibiotic tolerance may allow ivacaftor-induced SCVs to better persist in the CF lung when ivacaftor is used.

By evaluating the effects of ivacaftor on *S. aureus* infection in the CF rat and examining the potential effects of ivacaftor exposure on SCV formation and persistence, this work provides a basis for understanding why *S. aureus* continues to colonize the CF lung after the introduction of HEMT. Our results show that *S. aureus* is capable of surviving ivacaftor treatment in the CF rat, aligning with clinical studies that report persistent colonization of *S. aureus* in pwCF on modulators.^7–9^ Examination of *S. aureus* response to ivacaftor *in vitro* revealed the formation of ivacaftor-tolerant SCVs, which may be better suited to survive in the CF lung. Because these SCVs are capable of reversion to NCVs in the absence of ivacaftor, it is possible that similar SCVs may also be generated *in vivo*, but are not detected during clinical culture. Though additional studies are required to determine whether SCVs play a role in *S. aureus* persistence in pwCF, this work highlights the importance of examining bacterial adaptation to HEMT and the potential role of SCVs in persistent infection.
